# Vitamin C and N-acetylcysteine promote bovine adipose-derived mesenchymal stem cell proliferation and differentiation via Akt/mTOR/P70S6K signalling pathway for cultured meat production

**DOI:** 10.5713/ab.24.0776

**Published:** 2025-05-19

**Authors:** Sajida Naseem, Mei-Fu Xuan, Huan Hua, Sungkwon Park, Abid Manzoor, Hailong Teng, Huaina Jin, Xiangzi Li, Qiang Li

**Affiliations:** 1 Engineering Research Center of North-East Cold Region Beef Cattle Science & Technology Innovation, Ministry of Education, Department of Animal Science, Yanbian University, Yanji, China; 2 Department of Food Science and Biotechnology, Sejong University, Seoul, Korea

**Keywords:** Bovine Adipose-derived Mesenchymal Stem Cells, Cultured Meat, Oxidative Stress, Proliferation, Senescence

## Abstract

**Objective:**

Traditional meat production is insufficient to meet the increasing protein requirements, necessitating cultured meat, which is safe, worthwhile, and scalable. Fat is essential for making cultured meat more acceptable to consumers by enhancing flavour and providing a natural appearance. Mesenchymal stem cells from adipose tissue are a promising source for this purpose, but *in vitro* expansion of cells decreases their proliferation ability and increases cellular senescence. The objective of this study was to improve the proliferation and differentiation abilities of adipose-derived mesenchymal stem cells (AD-MSCs).

**Methods:**

In this study, vitamin C (VC) and N-acetylcysteine (NAC) antioxidants were used to treat AD-MSCs from Yanbian cattle testicles. Cell counting kit, 5-ethynyl-2’-deoxyuridine staining, reverse transcription quantitative polymerase chain reaction, and western blot were used to test the cell viability and proliferation ability of AD-MSCs, ORO staining, triglycerides assay, and adipogenic specific markers expression were determined to analyse the adipogenic differentiation ability. Furthermore, oxidative stress parameters and activation of the Akt/mTOR/P70S6K signaling pathway were also studied.

**Results:**

Results showed that VC and NAC both increased proliferation and differentiation ability of AD-MSCs by increasing the expression of cell cycle regulatory and differentiation genes and proteins expression, and decreasing the expression of cell cycle inhibitory factors, and up-regulating stemness markers expression, while co-treatment showed enhanced effect. Oxidative stress was reduced by decreased reactive oxygen species production, malondialdehyde content, and enhanced glutathione activity, as well as declined cellular senescence. Subsequently, the Akt/mTOR/P70S6K signaling pathway was activated by VC and NAC+VC treatment in AD-MSCs, while NAC only activated Akt expression, indicating its role in controlled cell growth.

**Conclusion:**

This research concludes that NAC (2 mM) and VC (200 μM) improved the proliferation, differentiation potential, and stemness by decreasing oxidative stress and senescence, parallelly activating Akt/mTOR/P70S6K signaling pathway, while combined treatment (NAC+VC) enhanced these effects, providing bases for their utilisation to culture fat in cultivated meat production.

## INTRODUCTION

The traditional system for livestock production has been a 15%–18% contributor to anthropogenic greenhouse gas emissions, a major factor impacting the environment, as estimated by the United Nations Food and Agriculture Organization [1,2]. As the human population increases, meat production is projected to increase 1.8-fold by 2050, further threatening the environment and public health [3]. Based on current industry estimates, cultured meat production requires fewer resources and less time compared to conventional meat production due to the exponential proliferation of cells *in vitro* and it is also environment friendly [4]. To meet rising protein demands, alternative meat and protein products are necessary and must replicate the texture, taste, and sensory characteristics of traditional meat to satisfy consumer expectations.

While current innovations in cellular and plant-based meat analogues have predominantly focused on replicating the myogenic component, fat remains a pivotal constituent that significantly enhances flavour, juiciness, texture, and overall sensory characteristics. Frank et al [5] showed that flavour volatiles were significantly higher in beef samples with over 10% intramuscular fat content, suggesting that the lipid content enhances beef flavour. Incorporating adipocytes into plant-based or muscle-based meat provides it with an animal-specific meat essence, as the aroma of meat during cooking results from the degradation of lipid content by heat [6]. Therefore, utilising adipocytes in culture or plant-based meats to completely exhibit the perfect profile of conventional meat. In a previous study, bovine adipose-derived mesenchymal stem cells (AD-MSCs) were used to prepare fat-rich edible meat tissue [7].

For cultured fat preparation, a significant amount of AD-MSCs are required, and more effective ways to expand these cells into a sufficient proportion are needed. However, *in vitro* expansion of AD-MSCs has shown limited proliferation ability due to an imbalance in redox status of stem cells [8,9], which leads to oxidative stress and decreased viability, proliferation, and differentiation of stem cells [10]. Furthermore, increased reactive oxygen species (ROS) levels cause DNA fragmentation, damage to cell membranes, and ultimately cell death. Therefore, reducing ROS is essential to enhance the proliferation and viability of stem cells in cultured fat and meat production.

It was found that antioxidants inhibit cellular senescence and maintain stemness of AD-MSCs by reducing ROS production during long-term *in vitro* expansion [8]. Vitamin C (VC), a well-known antioxidant, is involved in the three lines of defense system of living cells: scavenging free radicals, biosynthesis and activation of antioxidant enzymes, and repair of oxidative damage [11,12]. Treatment of replicatively senescent human bone marrow mesenchymal stromal cells after long-term culture with the antioxidant VC resulted in the elimination of excess ROS and partial restoration of the levels of antioxidant enzymes (catalase, SOD1 and 2, p-FOXO1, and p53) [13]. N-acetylcysteine (NAC), the N-acetyl derivative of the naturally occurring amino acid L-cysteine, is a precursor to cellular antioxidant enzyme glutathione [14]. In previous studies, NAC treatment of human dental follicle stem cells improved the proliferation rate, decreased stem cell senescence, and demonstrated a protective effect on induced pluripotent stem cells against oxidative stress [15,16]. It was found that the combination of *ex vivo* antioxidant treatment with NAC and ascorbic acid-2-phosphate increased the paracrine responsiveness of mouse bone marrow mesenchymal stem cells to diabetic wounds [17]. However, the effects of VC and NAC on Yanbian cattle AD-MSCs proliferation, differentiation, ROS inhibition, and senescence have not been reported. Yanbian cattle, renowned as one of China’s top five cattle breeds for its high-quality, tender meat with even marbling and rich flavour, undergoes castration to enhance beef quality, marbling, and tenderness. However, the removed testicles are globally discarded despite their potential as a valuable source of AD-MSCs [18].

This study aimed to investigate the effects of exogenous antioxidants, VC and NAC, on the proliferation and maintenance of cell function of Yanbian bovine AD-MSCs. The effects of these antioxidants on the proliferation rate, cell cycle regulators, stemness maintenance, differentiation capacity, and mitigation of cellular aging of bovine AD-MSCs under *in vitro* conditions were analysed, as well as their effects on proliferation-related signalling pathway was also examined. In this study, for the first time, we extracted AD-MSCs from Yanbian cattle testicles, which are otherwise wasted after castration. In conclusion, this research provides new theoretical basis to enhance the proliferation and differentiation potential of bovine AD-MSCs in extensive tissue cultivation, ultimately contributing to cultured meat production.

## MATERIALS AND METHODS

### Adipose-derived mesenchymal stem cells isolation from bovine testicles

**Figure 1:**
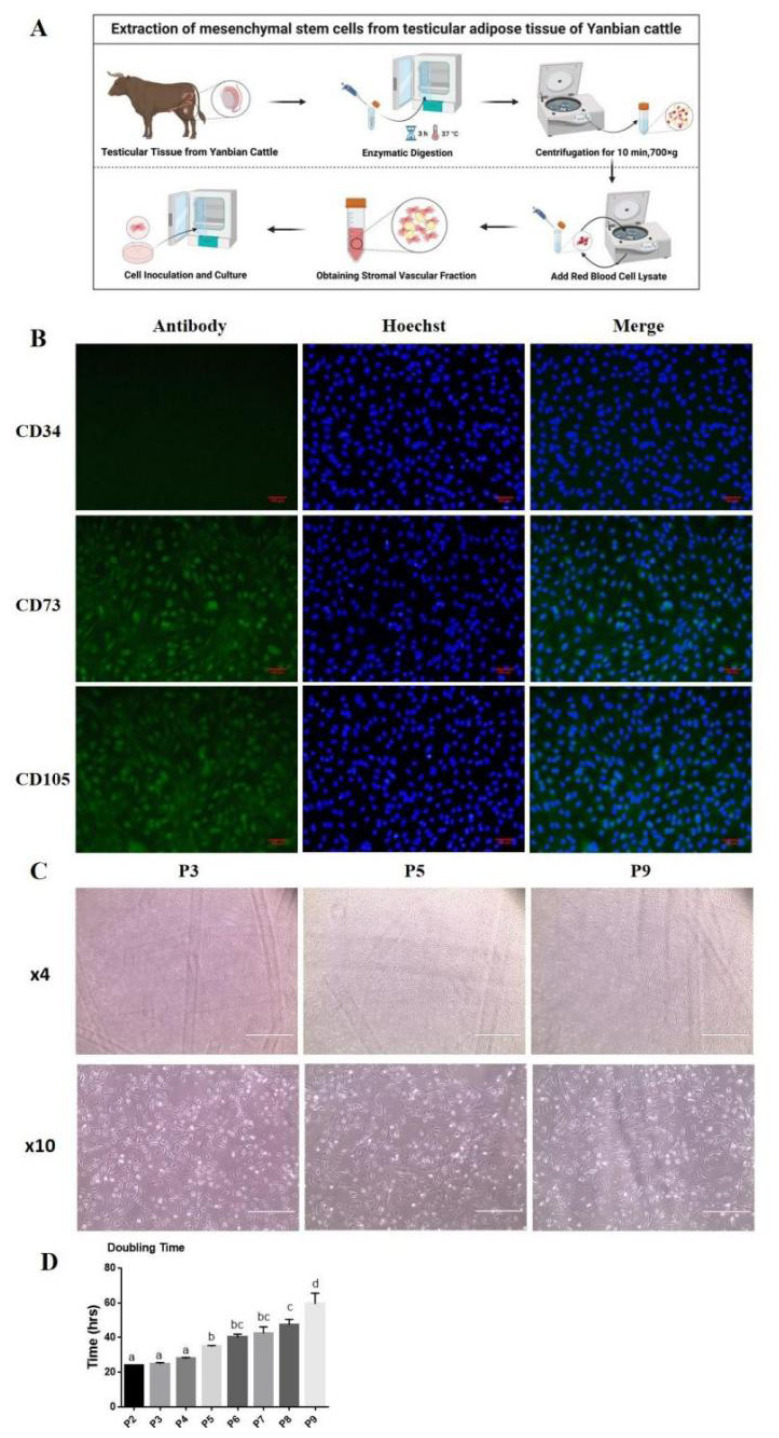
Identification of adipose derived mesenchymal stem cells (AD-MSCs) cell surface markers and morphology. (A) AD-MSCs extraction. (B) Immunofluorescence analysis showed that AD-MSCs expressed CD73 and CD105 and lacked CD34 expression. (C) Cell morphological characteristics during long-term expansion. Magnification “×4” with scale bar = 1,000 μm, and magnification “×10” with scale bar = 400 μm. (D) Cell doubling time was increased as passage number increased. All numerical data were shown as mean±SD; n = 3, p<0.05. ^a–d^ Values with a different letter above column differ significantly (p<0.05) and same or no superscript means no significant difference (p>0.05). SD, standard deviation.

Bovine AD-MSCs were extracted from adipose tissues of Yanbian cattle testicles using the collagenase type I (Biosharp Life Sciences, Beijing, China) digestion method [19]. Briefly, tissue segments were sterilized, digested enzymatically, and the filtrates were centrifuged at 700×g for 10 min. After centrifugation, the supernatants were aspirated, cells were seeded in standard medium and non-adherent cells were discarded. AD-MSCs were cultured in 100-mm plates in Dulbecco’s modified Eagle’s medium (DMEM, Gibco, Thermo Fisher Scientific, Suzhou, China) supplemented with 10% fetal bovine serum (FBS; Vivacell Biosciences, Shanghai, China) and 1% penicillin-streptomycin-gentamicin (Beyotime, Shanghai, China) in a cell culture incubator at 37°C with 5% CO_2_ (Figure 1A).

### Bovine adipose-derived mesenchymal stem cells characterization and serial passaging

The isolated primary cells reached 70%–80% confluency, were further passaged, and cell surface markers were identified by immunofluorescence assay at passage 3. The expression of two positive surface markers CD73 and CD105 and one negative marker CD34 was assayed. Briefly, cells were washed and fixed with 4% formaldehyde for 20 min; washed three times with PBS and permeabilized with 0.2% Triton X-100 for 30 min at room temperature. After washing, cells were blocked by 5% bovine serum albumin (Solarbio Life Sciences, Beijing, China) for 1 h on a shaker. Subsequently, cells were incubated overnight at 4°C with primary antibodies diluted in blocking solution. The cells were then washed three times with PBS and incubated with secondary antibodies for 2 hours at room temperature. Finally, the cells were incubated with hoechst for nuclear staining and after washing with PBS, photographed using a fluorescence microscope (WF10X; Olympus, Tokyo, Japan). Antibodies used were as follows: CD34 (bs-0646R), CD73 (bs-4834R), and CD105 (bs-0579R) primary antibodies (Bioss antibodies, Beijing, China) and goat anti-rabbit IgG H&L/FITC (bs-0295G-FITC) secondary antibody; all antibodies dilutions were 1:200.

To check the proliferation ability and morphological changes in serial passaging of AD-MSCs, passage 2 (P2) cells were cultured in 100 mm dish with 10 mL of growth medium, and after every 48 hours cells were collected by trypsinization, counted, and further passaged up to passage 9 (P9). AD-MSCs doubling time was calculated and morphological characteristics were observed.

### Adipose-derived mesenchymal stem cells treatment and proliferation activity assays

AD-MSCs were cultured in 96-, 24-, 12-, or 6-well plates according to experimental requirements, and treated with different concentrations of VC (CAS. 50-81-7) and NAC (CAS. 616-91) (Sigma-Aldrich, St. Louis, MO, USA) for dose determination (Supplements 1–3). Cell counting kit (CCK)-8 and 5-ethynyl-2’-deoxyuridine (EdU) staining assays were used to assess cell viability and proliferation activity. Briefly, cells were passage in 96 wells plate at a density of 3,000 cells/well. After attachment, the cells were treated with NAC (2 mM), VC (200 μM), and NAC+VC (2 mM+200 μM) in 100 μL medium per well. After 24, 48, 72, and 96 h of treatment, CCK-8 kit (APExBIO, Shanghai, China) was used according to the manufacturer’s instructions and cell activity was measured at 450 nm using a microplate reader (De Tie, HBS-1096A Microplate reader). Afterwards, the proliferative activity of bovine AD-MSCs was detected by EdU staining according to the manufacturer’s instructions after 48 h (RIBOBIO, Guangzhou, China).

### Extraction of RNA and real-time quantitative polymerase chain reaction

Total RNA was extracted using TRIzol reagent (Invitrogen, Waltham, MA, USA; Thermo Fisher Scientific) coherent to manufacturer’s directions. Complementary DNA was synthesised using the FastKing One Step kit (Tiangen Biotechnology, Beijing, China) from 1 μg of RNA from each sample as described by manufacturer. To measure the quantities of genes of interest relative to glyceraldehyde-3-phosphate dehydrogenase (GAPDH) quantity in different treatments, real-time quantitative polymerase chain reaction (PCR) was used. Super Real PreMix Plus (SYBR Green, Tiangen Biotechnology) with appropriate forward and reverse primers (Table 1) was used to quantify gene expression (Agilent Fast Real-Time PCR System), and thermal cycling was performed as mentioned by manufacturers of the kit. The mRNA expressions of genes were analysed by relative quantification method (2^−ΔΔCt^).

### Western blotting

Cell cycle regulatory proteins and Akt/mTOR/P70S6K pathway-related proteins were blotted. Radioimmunoprecipitation analysis buffer with 1% phenyl methyl sulfonyl fluoride (PMSF; Beyotime) was used for cell lysis to extract protein. Cell lysates were collected and centrifuged at 4°C, at 14,000×g for 15 min and supernatant were collected for protein concentration measurement by Pierce BCA Protein Assay Kit (23227, Thermo Fisher Scientific). After protein concentration determination, samples were diluted with 5x loading buffer and 50 μg of protein from every sample were separated on 8%–12% sodium dodecyl sulfate polyacrylamide gels (Solarbio, Beijing, China). Membranes were washed and overnight incubated on a shaker at 4°C with primary antibodies (CDK1 [bs-0542R], CDK2 [bs-10726R], P21 [bs-0741R], β-actin [bs-0061R], mTOR [bs-1992R], p-mTOR [bs-3494R], P70S6K [bs-3498R], and p-P70S6K [bs-3498R] with 1:1,000 dilution; AKT [bs-10724R], p-AKT [bs-0876R] with 1:2,000 dilution) (Bioss Antibodies, Beijing, China). Followed by incubation with secondary antibodies (HRP-labeled goat anti-rabbit IgG [H+L] [A0208] 1:1,000; HRP-labeled goat anti-mouse IgG [H+L] [A0216] 1:1,000 [Beyotime]) for 2 hours at room temperature. Enhanced chemiluminescence (ECL; Beyotime) was used to visualise the protein bands and results were analysed using an Azure 600 multifunctional imaging analyzer. ImageJ software was used to quantify protein bands and β-actin bands were used as loading control for normalisation.

### Adipose-derived mesenchymal stem cells differentiation analysis

For AD-MSCs adipogenic differentiation, 2% horse serum was used with 1% penicillin-streptomycin, supplemented with 100 μM palmitoleic acid in each treatment and control group. AD-MSCs were differentiated for 96 h, and an Oil Red O staining kit (G1262, Solarbio) was used to assess the lipid droplet formation and triglyceride content (Applygen Technologies, Beijing, China) was assayed according to manual instruction.

### Senescence and oxidative stress parameters analysis

AD-MSCs were culture in 6 well plates and after treatment with VC and NAC, senescence and oxidative stress-related parameters were assayed. β-galactosidase assay was used to detect the cellular senescence of AD-MSCs at P5 and P9 after treatment [19]. ROS kit was used to detect ROS production [20]. Lipid peroxidation was determined by measuring malondialdehyde (MDA) content and glutathione peroxidase (GPx) activity level was also assayed. All experiments were done according to manufacturer’s instructions and all kits were purchased from Beyotime.

### Data statistics and analysis

GraphPad Prism 6.07 (GraphPad Software, San Diego, CA, USA) was used for one-way analysis of variance and Tukey’s multiple comparisons test to determine the significant difference between treatments at significance level of p<0.05. All experiments were done in triplicates and the data were presented as mean±standard deviation.

## RESULTS

### Bovine adipose-derived mesenchymal stem cells characterization and morphological changes during serial passaging

Immunofluorescence analysis revealed that AD-MSCs extracted from Yanbian cattle showed high expression CD73, CD105 and lack the expression of CD34 cell surface marker (Figure 1B). It is crucial to analyse progressive changes in the morphology and proliferation ability of AD-MSCs during serial passaging to obtain a large number of cells *in vitro*. For this purpose, AD-MSCs were continuously cultured and passaged up to P9, morphological changes were observed, and the cell doubling time was calculated at each passage. In early passages cells showed spindle and fibroblast-like structures, while as passage number increasing cells become more flattened and larger in size (Figure 1C). Cell doubling time increased significantly from passage 5 (P5) onward, showing progressive elongation through P9 (Figure 1D).

### Proliferation and stemness of adipose-derived mesenchymal stem cells enhanced by vitamin C and N-acetylcysteine

**Figure 2:**
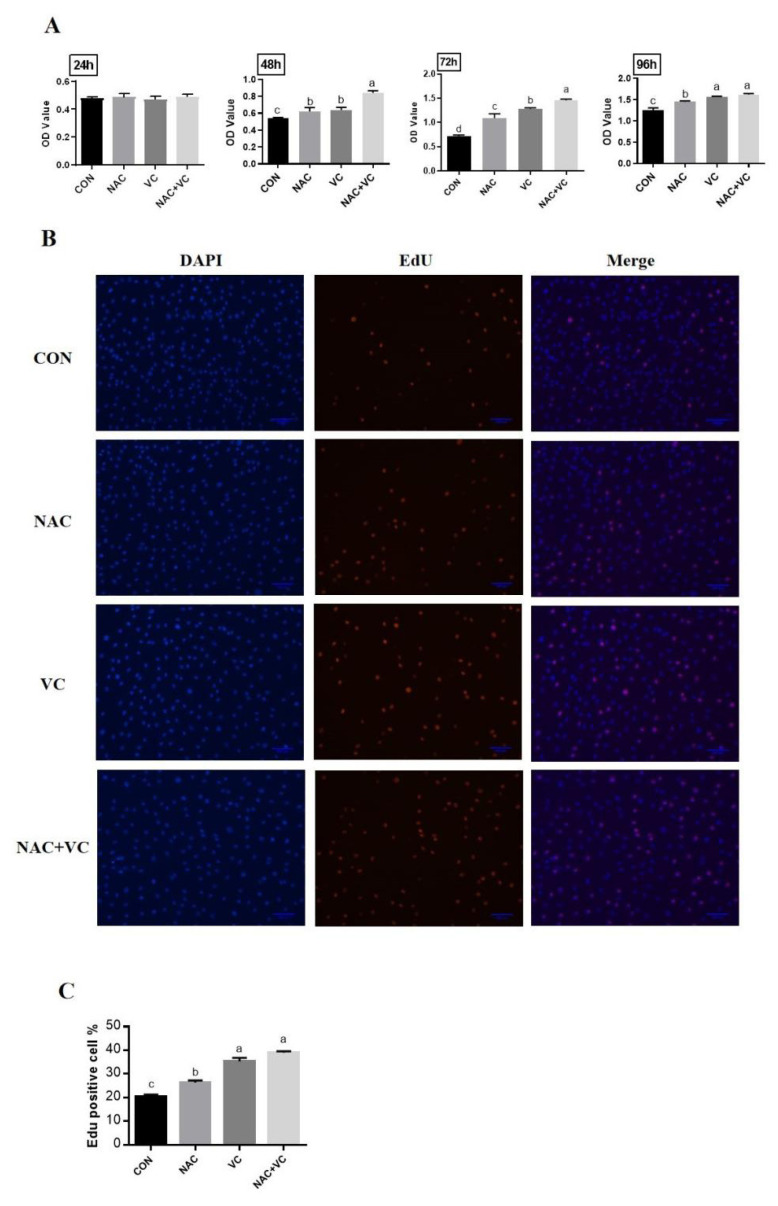
Combined effect of vitamin C (VC) and N-acetylcysteine (NAC) on adipose derived mesenchymal stem cells (AD-MSCs) proliferation. (A) Cell viability OD value; AD-MSCs were treated with 0 (CON), 2 mM of NAC, 200 μM of VC and NAC+VC (2 mM+200 μM) for 24, 48, 72, 96 hours. (B) EdU staining; AD-MSCs were treated with 0 (CON), 2 mM of NAC, 200 μM VC and NAC+VC (2 mM+200 μM). Magnification is ×10, scale bar = 200 μm. (C) EdU-positive cell percentage. All numerical data were shown as mean±SD; n = 3, p<0.05. ^a–d^ Values with a different letter above column differ significantly (p<0.05) and same or no superscript means no significant difference (p>0.05). EdU, 5-ethynyl-2’-deoxyuridine; SD, standard deviation.

The combined effect of VC and NAC on cell viability was analysed and the results indicated that viability of cells was higher at 72 and 96 h than that in the control group (p<0.05) (Figure 2A). After adding 2 mM NAC and 200 μM VC to treat Yanbian bovine adipose MSCs for 48 h, microscopic examination revealed that the number of cells increased and the cell growth status improved under the treatment conditions of NAC and VC, compared with that of the CON group (Supplement 1). EdU staining of Yanbian bovine adipose MSCs after 48 h of treatment with 2 mM NAC, 200 μM VC, and NAC+VC were observed under a microscope, revealing increased EdU positive cell percentages of 26%, 35%, and 39%, respectively, as compared to the control (20%). The NAC+VC and VC treated cells showed a high rate of EdU-positive cells compared to the NAC and control groups (Figures 2B, 2C).

**Figure 3:**
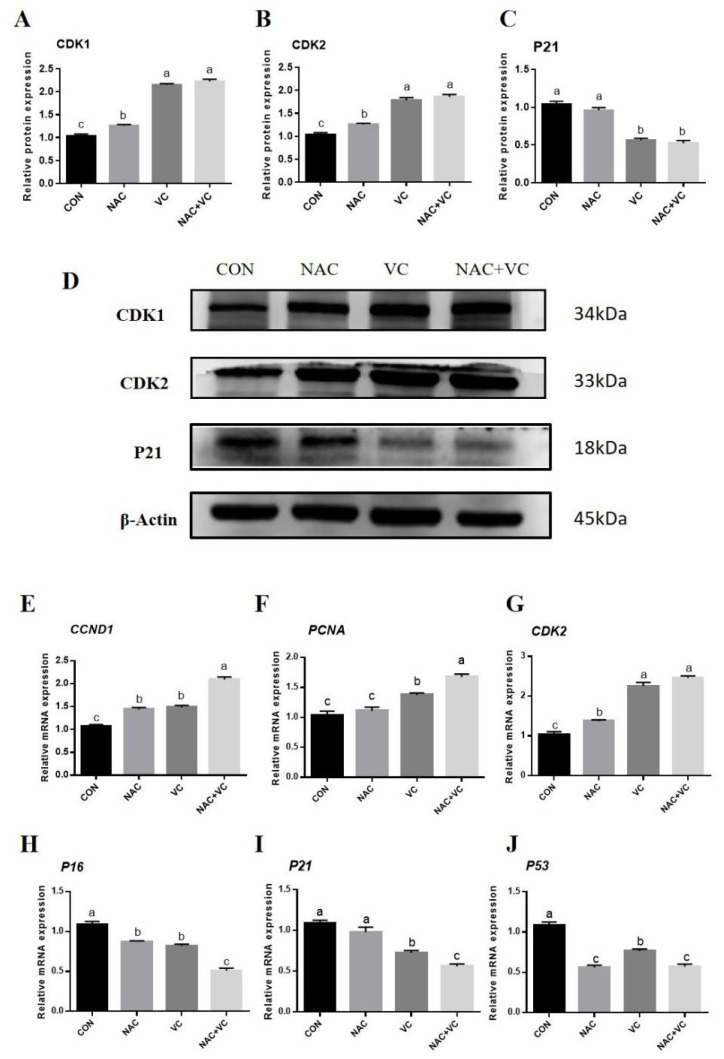
Effect of vitamin C (VC) and N-acetylcysteine (NAC) on adipose derived mesenchymal stem cells (AD-MSCs) cell cycle regulatory proteins and genes expression. AD-MSCs were treated with 0 (CON), 2 mM of NAC, 200 μM VC and NAC+VC (2 mM+200 μM). (A–D) Western blot analysis; Relative protein expression of CDK1, CDK2 and P21. (E–J) RT-qPCR analysis; Relative mRNA expression of CCND1, PCNA, CDK2, P16, P21, P53. All numerical data were shown as mean±SD; n = 3, p<0.05. ^a–c^ Values with a different letter above column differ significantly (p<0.05) and same or no superscript means no significant difference (p>0.05). RT-qPCR, reverse transcription quantitative polymerase chain reaction; SD, standard deviation.

To explore the combined effects of VC and NAC on cell cycle regulatory factors, Yanbian bovine AD-MSCs were treated with 200 μM VC, 2 mM NAC, and 200 μM VC+2 mM NAC for 96 h. Western blotting results showed that the NAC+VC co-treatment group had the highest CDK1 and CDK2 protein expression as compared to the control group, while no significant difference with the VC group. Compared with the CON and NAC groups, P21 protein expression was significantly lower in both the VC and co-treatment groups (p<0.05) (Figures 3A–3D).

The reverse transcription quantitative polymerase chain reaction (RT-qPCR) analysis of proliferation genes was carried out after 72 h of AD-MSCs treatment, and the results showed that mRNA expression of *CCND1* and *PCNA* gene was significantly higher in all treatments, except expression of *PCNA* in NAC treated group as compared to control, while both gene mRNA was highest in NAC+VC group (p<0.05) (Figures 3E, 3F). The *CDK2* mRNA expression was significantly leading in VC and NAC+VC groups (p<0.05) than in all other treatment groups (Figure 3G). The gene expression levels of cycle inhibitory factors (*P53*, *P16* and *P21*) were also analysed, and the expression levels of *P16* and *P21* were significantly reduced in all treatment groups, while the combined treatment group (NAC+VC) exhibited significant decrease (p<0.05), and no significant difference was found in *P21* when treated with NAC compared to control (p<0.05) (Figures 3H, 3I). Gene expression of *P53* was also decreased compared to that in the control group; however, NAC and NAC+VC groups showed similar expression (Figure 3J). Combined treatment with VC and NAC not only decreased the expression of cell cycle inhibitory factors, but also enhanced the expression of cell cycle regulatory genes and proteins, consequently improving AD-MSCs proliferation.

**Figure 4:**
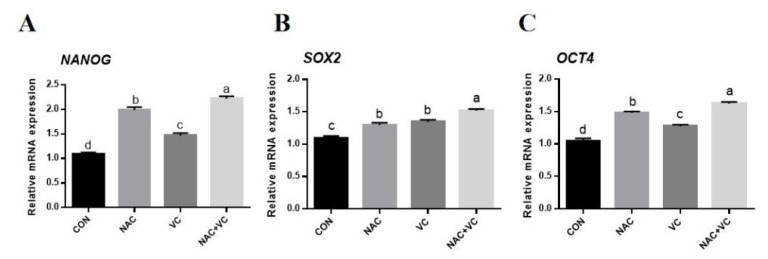
Combined effect of vitamin C (VC) and N-acetylcysteine (NAC) on adipose derived mesenchymal stem cells (AD-MSCs) stemness gene expression. AD-MSCs were treated with 0 (CON), 2 mM of NAC, 200 μM VC and NAC+VC (2 mM+200 μM). (A–C) Relative protein expression of NANOG, SOX2, and OCT4. All numerical data were shown as mean±SD; n = 3, p<0.05. ^a–d^ Values with a different letter above column differ significantly (p<0.05) and same or no superscript means no significant difference (p>0.05). SD, standard deviation.

The expression levels of stemness genes in adipose MSCs in the different treatment groups were also detected by RT-qPCR. Compared to the CON group, the expression of *NANOG*, *SOX2*, and *OCT4* mRNA in each treatment group was significantly higher (p<0.05) (Figures 4A–4C). The *NANOG* mRNA expression was significantly highest in NAC+VC treatment group, followed by the NAC and VC groups as compared to control, whereas *OCT4* also revealed a similar expression pattern as *NANOG*. The NAC+VC treatment group revealed the highest expression of *SOX2* compared to the control (p<0.05), while no significant difference was observed between NAC and VC treatments.

### Adipogenic differentiation of adipose-derived mesenchymal stem cells improved by vitamin C and N-acetylcysteine

**Figure 5:**
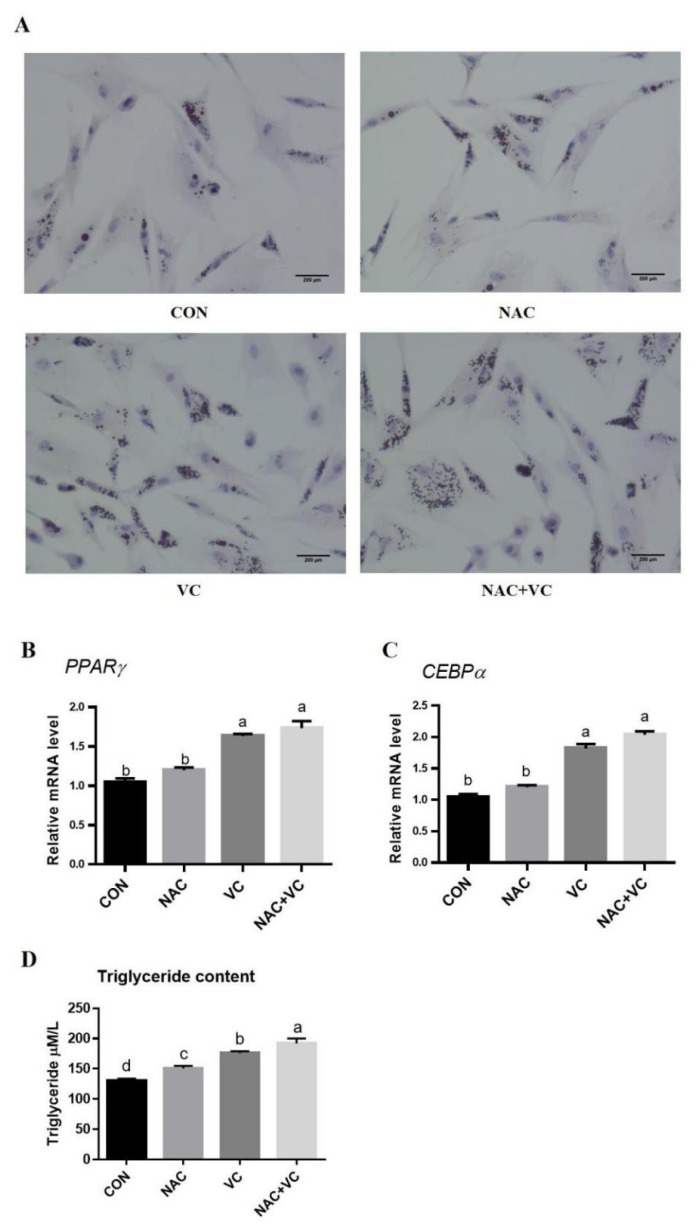
Effect of vitamin C (VC) and N-acetylcysteine (NAC) on adipose derived mesenchymal stem cells (AD-MSCs) differentiation. (A) Oil Red O staining of AD-MSCs adipogenic differentiation (bar = 200 μm). (B, C) Relative mRNA expression of PPARγ and C/EBPα. (D) Triglycerides content. All numerical data were shown as mean±SD; n = 3, p<0.05. ^a–d^ Values with a different letter above column differ significantly (p<0.05) and same or no superscript means no significant difference (p>0.05). SD, standard deviation.

Differentiated cells were observed by microscopic examination following Oil Red O staining (Figure 5A). The NAC group showed more lipid droplets as compared to the CON group, whereas the VC group and the NAC+VC group showed a great number of lipid droplets. Differentiation related genes such as *PPARγ* and *C/EBPα* showed a significant difference in VC and NAC+VC treated groups compared to control group (p<0.05) indicating their ability to improve the AD-MSCs differentiation (Figures 5B, 5C). Triglyceride content was significantly increased by NAC+VC, followed by VC and NC treatment as compared to control (Figure 5D).

### Senescence and oxidative stress decreased through vitamin C and N-acetylcysteine

**Figure 6:**
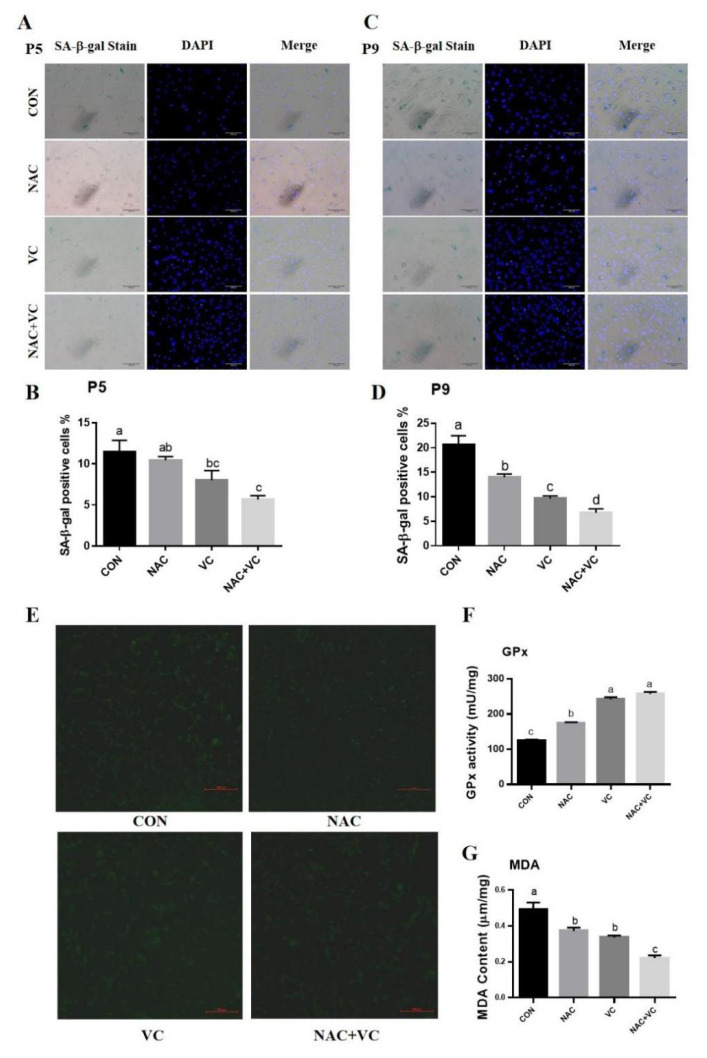
Effect of vitamin C (VC) and N-acetylcysteine (NAC) on adipose derived mesenchymal stem cells (AD-MSCs) senescence and oxidative stress related parameters. (A–D) β-galactosidase staining; P5 and P9 AD-MSCs were treated with 0 (CON), 2 mM of NAC, 200 μM VC and NAC+VC (2 mM+200 μM). Magnification ×20, scale bar = 200 μm. (E) Reactive oxygen species (ROS) staining. Magnification ×4, scale bar = 500 μm. (F) Total glutathione peroxidase (GPx) activity. (G) Malondialdehyde (MDA) content. All numerical data were shown as mean±SD; n = 3, p<0.05. ^a–d^ Values with a different letter above column differ significantly (p<0.05) and same or no superscript means no significant difference (p>0.05). SD, standard deviation.

Cellular senescence in each treatment group was observed under bright-field microscopy, and the nuclei were stained to analyse the percentage of senescence in both P5 and P9 treated AD-MSCs. Senescence was increased in P9 control cells as compared to P5 control, while all treatments tremendously ameliorated senescence as compared to control at both P5 and P9, especially NAC+VC treatment (p<0.05) (Figures 6A–6D).

Intracellular oxidative stress in the different treatment groups was detected by ROS staining. The ROS content in all treatment groups was significantly reduced, NAC and VC treatment reduced the ROS content to the same extent. In addition, combined treatment showed a great reduction in ROS as compare to control (p<0.05) (Figure 6C). To clarify the effects of NAC and VC on intracellular total GPx activity and damage by lipid peroxidation, GPx activity and MDA levels were assayed. The results showed that GPx activity was significantly higher (p<0.05) (Figure 6D) and MDA level was significantly lower (p<0.05) (Figure 6E) in all treatment groups than in the control group. GPx activity was significantly enhanced in the VC treated and NAC+VC co-treated groups, compared to NAC-treated group (p<0.05), and MDA levels were significantly lower in the NAC+VC treated group than in the NAC and VC separate treatment groups (p<0.05).

### Akt/mTOR/P70S6K pathway activation by vitamin C and N-acetylcysteine

**Figure 7:**
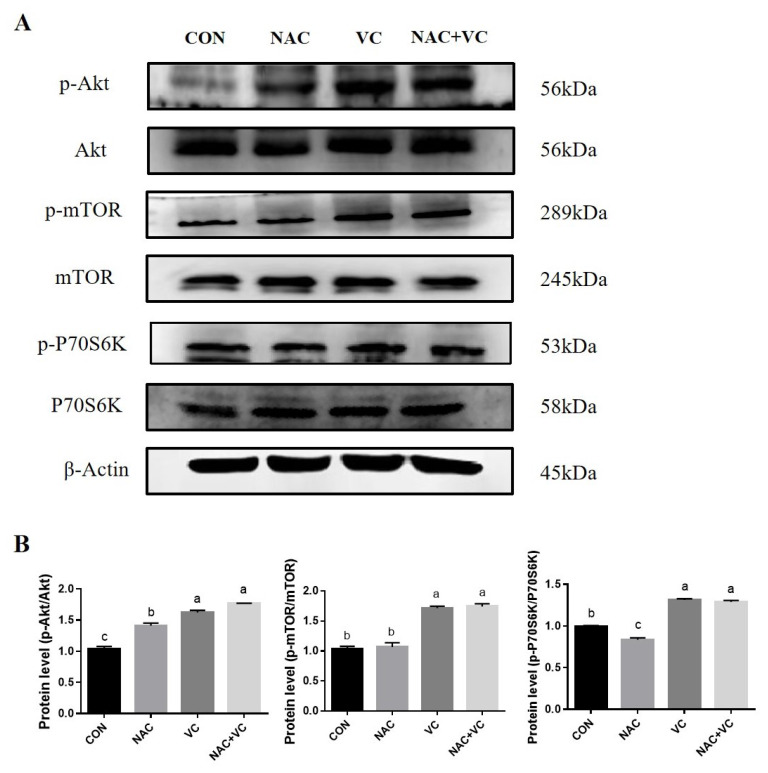
Effect of vitamin C (VC) and N-acetylcysteine (NAC) on Akt/mTOR/P70S6K pathway activation in adipose derived mesenchymal stem cells (AD-MSCs). Western blot analysis; relative protein expression of Akt, p-Akt, m-TOR, p-mTOR, P70S6K and p-P70S6K. All numerical data were shown as mean±SD; n = 3, p<0.05. ^a–c^ Values with a different letter above column differ significantly (p<0.05) and same or no superscript means no significant difference (p>0.05). SD, standard deviation.

In order to explore the effects of NAC and VC on the mTOR pathway in Yanbian bovine AD-MSCs, the expression of Akt/mTOR/P70S6K signalling pathway related proteins was detected by western blotting. Compared with the CON group, the VC and the combined (NAC+VC) groups showed significantly increased expression of p-Akt, p-mTOR and p-P70S6K proteins (p<0.05) (Figure 7), suggesting that VC and NAC+VC may regulate the proliferation and differentiation of Yanbian bovine AD-MSCs through activating the Akt/mTOR/P70S6K signalling pathway. NAC activates Akt protein; however, whether it affects the mTOR pathway requires further investigation.

## DISCUSSION

As a critical component for cultured meat quality, fat contributes to sensory attributes, and AD-MSCs, known for their abundance in adipose tissue, hold significant potential for stem cell-based fat and meat production [11,21]. Although AD-MSCs have been widely utilized for cultured fat production, the high demand for seed cells remains a significant barrier to their large-scale application [22]. Moreover, long-term *in vitro* passaging decreases the proliferation and differentiation capacities of cells and causes cellular senescence [19]. Therefore, it is important to enhance the proliferation and differentiation abilities of cells to utilize them for cultured fat production by optimizing media formulation or growth promoting substances [23,24]. In this study, we found that VC and NAC improved the proliferation and adipogenic differentiation of AD-MSCs by decreasing oxidative stress and senescence through the activation of the Akt/mTOR/P70S6K pathway.

In cattle farming, castration is routinely performed to improve meat quality [25], and the removed testicles are typically discarded as biological waste. However, our study demonstrated that adipose tissue isolated from the testes contained adipose stromal cells capable of differentiating into adipocytes; Bräunig previously extracted MSCs from mice omentum and apididymis fat depots [26]. Notably, the encapsulation of adipose tissue within the testes minimises the risk of contamination during primary cells isolation. To further characterise these stromal cells, we assessed the expression of surface markers that are commonly associated with AD-MSCs. CD73 and CD105 are recognised as positive surface markers for mesenchymal stem cells derived from various tissues, whereas CD34 is identified as a negative marker according to the minimal criteria for AD-MSCs established by the International Society for Cellular Therapy [27]. In this study, immunofluorescence analysis of AD-MSCs revealed high expression of CD73 and CD105 and no expression of CD34, confirming the cell type (Figure 1). For self-renewal capacity evaluation, AD-MSCs were continuously passaged, and the cell doubling time was calculated at each passage. The results showed an increase in cell doubling time with an increase in passage number, and the change in cell morphology was consistent with previous studies, which reported that adipose stem cells decreased their ability to proliferate and became more flattened in shape. Morphological changes in our study were clearly observed at P9 which was consistent with Song’s research, although inconsistent with Jin’s report at P15; this might be due to the variation in cell source and culture conditions [19,28].

VC is widely recognised for its antioxidant properties and crucial role in promoting cell proliferation by facilitating cell cycle progression through inhibiting cell cycle arrest at the G0/G1 phase, and increasing the expression of cell cycle regulatory genes (CDK1, CDK2, and Ki67) by activating the PI3K/AKT/mTOR pathway *in vitro* [29]. Similarly, NAC is a derivative of the sulfur-containing amino acid cysteine, which is converted to compounds such as glutathione, a key intracellular antioxidant essential for protecting cells from oxidative stress and promoting cell survival [19,30]. NAC has been shown to enhance cell proliferation, particularly following oxidative stress or exposure to toxic factors, and also improves long-term expansion of AD-MSCs [19]. VC and NAC both give a complementary effect by reducing oxidative and inflammatory stress, and apoptosis while promoting genes expression and pathways related to cell proliferation and differentiation [31,32]. In our experiment, CCK-8 and EdU assays confirmed that treatment with appropriate concentrations of VC and NAC significantly enhanced the proliferation of AD-MSCs, consistent with findings from previous research (Figure 2).

Cyclin-dependent kinases (CDKs), particularly CDK1 and CDK2, are crucial regulators of cell cycle progression, driving cells through key checkpoints like the G2/M transition [33]. The activity of these kinases is tightly controlled by inhibitors such as P21, which induces growth arrest by inhibiting CDK1 and CDK2 [34]. In this study, supplementation with VC and NAC significantly promoted the proliferation and cell cycle progression of bovine AD-MSCs by upregulating CDK1 and CDK2 expression while down-regulating P21, thereby suppressing the P53/P21 pathway, consistent with previous findings [31]. Interestingly, higher NAC concentrations elevated P21 levels, suggesting a dose-dependent effect in which NAC may induce cellular stress responses at elevated doses [35]. Additionally, markers like *PCNA* and cyclin D1 (*CCND1*), which are essential for DNA synthesis and G1/S phase transition, showed significant upregulation under VC and NAC treatment, indicating enhanced DNA replication and cell proliferation [36,37].

Moreover, stemness genes such as *NANOG*, *SOX2*, and *OCT4*, which are critical for maintaining pluripotency and self-renewal in stem cells, exhibited increased expression under VC and NAC treatment [38]. In our experiment, the combined treatment with VC and NAC most effectively promoted stemness by upregulating these genes (Figures 4A–4C). Mechanistically, VC likely modulates *NANOG* expression by regulating DNA methylation and activating the JAK/STAT pathway, whereas NAC may influence *SOX2* expression by maintaining GSH levels and related signalling pathways [39–41]. In addition, *OCT4* expression may be regulated by both antioxidants via ROS modulation and transcriptional networks [40,42]. The coordinated upregulation of both cell cycle-related and stemness-related genes is consistent with the observed enhancement in AD-MSCs proliferation and maintenance following VC and NAC treatment.

Further supporting these findings, the literature suggests that the combined application of VC and NAC significantly accelerates the differentiation of mesenchymal stem cells into adipocytes, promoting increased expression of adipocyte-specific genes such as *PPARγ* and *C/EBPα* [43,44]. In our study, the differentiation capacity of AD-MSCs treated with VC and NAC was notably improved, while the combined treatment yielded increased lipid droplet size and number, higher expression of adipogenic markers and increased triglyceride content. These results suggest that antioxidants can improve the efficiency of adipogenic differentiation in AD-MSCs by modulating differentiation-related signalling pathways.

VC and NAC treatment improves cellular stemness and reduces senescence, by reducing ROS levels and improving antioxidative capacity, which was further confirmed by SA-β-gal staining, ROS, GPx production and MDA content measurement analysis [45,46]. VC and NAC, both antioxidants, reduce intracellular oxidative and inflammatory stress which are important factors in slowing down the process of cellular senescence by scavenging intracellular free radicals, reducing oxidative stress, neutralising peroxidases, repairing DNA damage and inhibiting the formation of cellular senescence markers [17–20]. In this research, AD-MSCs senescence increased at P9 compared to P5 which is similar to the results of Liao et al [9] and Song et al [19], reported a significant increase in cellular senescence at P9. The number of cells in the antioxidant treated groups was higher than the control group, and the percentage of senescent cells was lower than the control group (Figures 6A–6D). The combined treatment of NAC and VC increased the intracellular GPx activity and reduced ROS production and MDA level more effectively compared to the individual treatment (Figure 6). This may be due to the combined antioxidant effects of NAC and VC because acetylcysteine acts as a precursor molecule for glutamine and is converted to glutathione, whereas VC can regenerate oxidised glutathione [46].

Moreover, we measured the protein expression of Akt, p-Akt, mTOR, p-mTOR, P70S6K and p-P70S6K to confirm the activation of AKT/mTOR/P70S6K signalling pathway by VC and NAC treatment. Akt (protein kinase B) signalling involves several pathways related to various biological functions like cell migration, proliferation, survival, and regeneration. mTOR is a protein kinase that plays a crucial function in regulating protein translation through p70S6K and protein degradation. The downstream effector of Akt is mTOR/p70S6K, which is also involved in cell growth and senescence [47,48]. VC treatment has been shown to reverse cellular senescence by reducing ROS through the activation of Akt/mTOR pathway in rat bone marrow MSCs [20]. Kashino et al [45] reported that AA had protective effects against cellular senescence via scavenging ROS. NAC, with its antioxidant properties, may affect the oxidative modification of AKT in the cellular signalling pathway, but did not activate mTOR/P70S6K pathway in this study, in contrast to a previous study which showed NAC at 100μM activate mTOR/P70S6K pathway in intestinal porcine epithelial cells, probably due to the concentration, culture conditions, or cell type differences [49]. The age of animal used as the cell source and cell type might have different effect on the proliferation and differentiation capacity with VC and NAC treatment, which needs to further verified. As this is the first study, to the best of our knowledge, on AD-MSCs extracted from cattle testicles, more studies should be conducted in the future to explore the differences between this cell type and cell source with others.

## CONCLUSION

In the current study, it was confirmed that combined treatment of NAC and VC activates Akt/mTOR/P70S6K pathway by improving cellular redox status and decreasing melondialdehyde production. In addition, optimal concentrations of 2 mM NAC and 200 μM VC significantly reduced cellular senescence even at passage 9 and enhanced AD-MSCs stemness, proliferation, and adipogenic differentiation gene expression, whereas combined treatment (NAC+VC) escalated these effects. Altogether, these data suggest the possible role of VC and NAC as supplements for large-scale cultured fat production for cultured meat.

## Figures and Tables

**Table 1:** Genes and primer sequences

Genes	Accession number	Sequence (5’–3’)	Product size
*GAPDH*	NSO_4761240	Forward: ACTCTGGCAAAGTGGATGTTGTC	95
		Reverse: GCATCACCCCACTTGATGTTG	
*CCND1*	NM_01034494.1	Forward: CTCGGTGTCCTACTTCAAGTGTGTG	143
		Reverse: TCGCAGACCTCCAGCATCCAG	
*PCNA*	NM_0010149.1	Forward: GTCCAGGGCTCCATCTTGAAGAAAG	76
		Reverse: GCTGCACCAAGGAGACATGAGAC	
*CDK2*	XM_036485921	Forward: ACGGAGCTTGTTATCGCAAATGC	146
		Reverse: AGGTACTGGCTTGGTCACATCTTG	
*P16*	NM 181024	Forward: TGCGAAGATCAGAGCGAAATACCC	80
		Reverse: CAGTGATGTCGGATGGAACCAGATAC	
*P21*	NM 176784	Forward: GTCCAGGGACGCGCATCAAATC	69
		Reverse: CAAAGTCGAAGTTCCACCGCTCTC	
*P53*	NM_205793.2	Forward: GCCCATCCTCACCATCATCACAC	225
		Reverse: GCACAAACACGCACCTCAAAGC	
*PPARγ*	NM_174693.2	Forward: ATCTGCTGCAAGCCTTGGA	108
		Reverse: TGGAGCAGCTTGGCAAAGA	
*C/EBPα*	NM_001015537.1	Forward: GGGCGGCATCTGCGAACAC	97
		Reverse: GCCAGGAACTCGTCGTTGAAGG	
*NANOG*	XM_024994376.1	Forward: CACTGTCTCTCCTCTTCCCTCCTC	104
		Reverse: TCTCTTCCTTCTCTGTGCTCTCCTC	
*OCT4*	NM_001083669.1	Forward: GCCACCATGGCGGGACAC	76
		Reverse: TCAGTTTGAATGCATAGGAGAGC	
*SOX2*	NM_181024	Forward: GCCACCATGTACAACATGATGG	69
		Reverse: TCACATGTGCGAGAGGGGCA	

## SUPPLEMENTARY MATERIAL

**Supplement 1**. Effect of of Vitamin C (VC) and N-acetylcyteine (NAC) on adipose derived mesenchymal stem cells (AD-MSCs) proliferation.

**Supplement 2**. Effect of Vitamin C (VC) and N-acetylcyteine (NAC) on adipose derived mesenchymal stem cells (AD-MSCs) cell cycle regulatory proteins.

**Supplement 3**. Morphology of adipose derived mesenchymal stem cells (AD-MSCs) treated with Vitamin C (VC) and N-acetylcyteine (NAC).
