# Supplementary figures and images for: Vitamin C and N-acetylcysteine promote bovine adipose-derived mesenchymal stem cell proliferation and differentiation via Akt/mTOR/P70S6K signalling pathway for cultured meat production
Source: Anim Biosci. 2025 May 19;38(10):2250–65. doi: 10.5713/ab.24.0776 (PMC12415370; doi:10.5713/ab.24.0776)

Supplement 1.

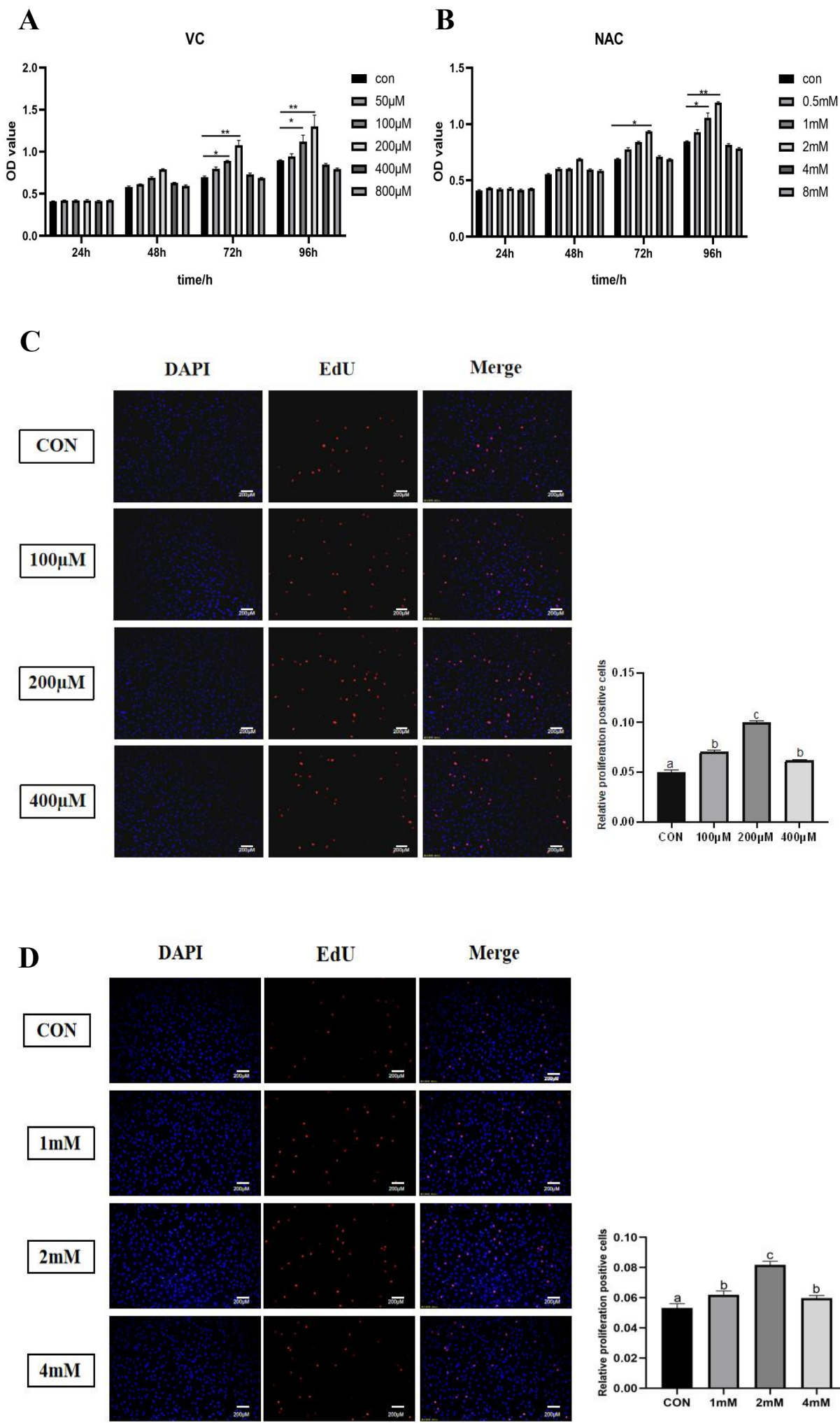

Supplement: Supplementary file 1 [file ab-24-0776-Supplementary-1.pdf]

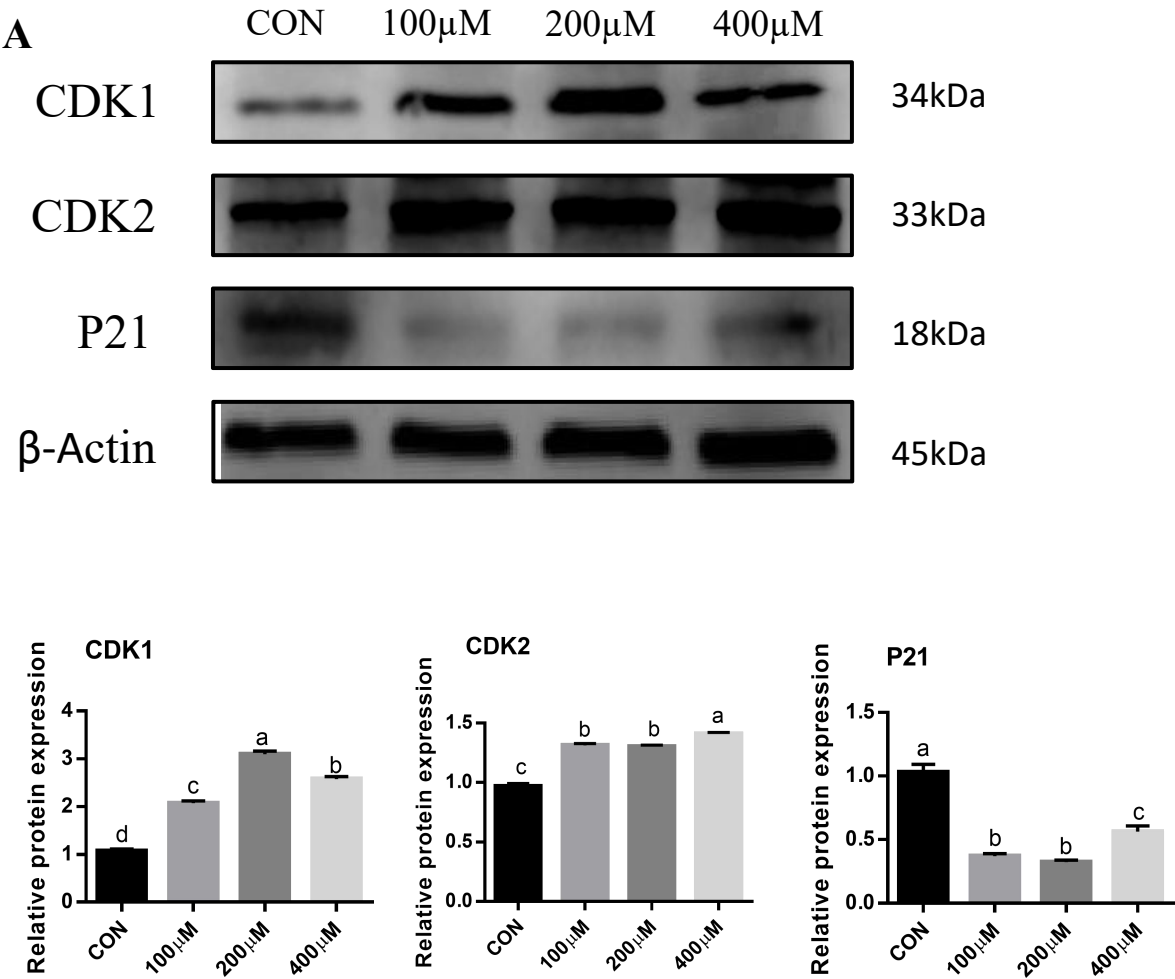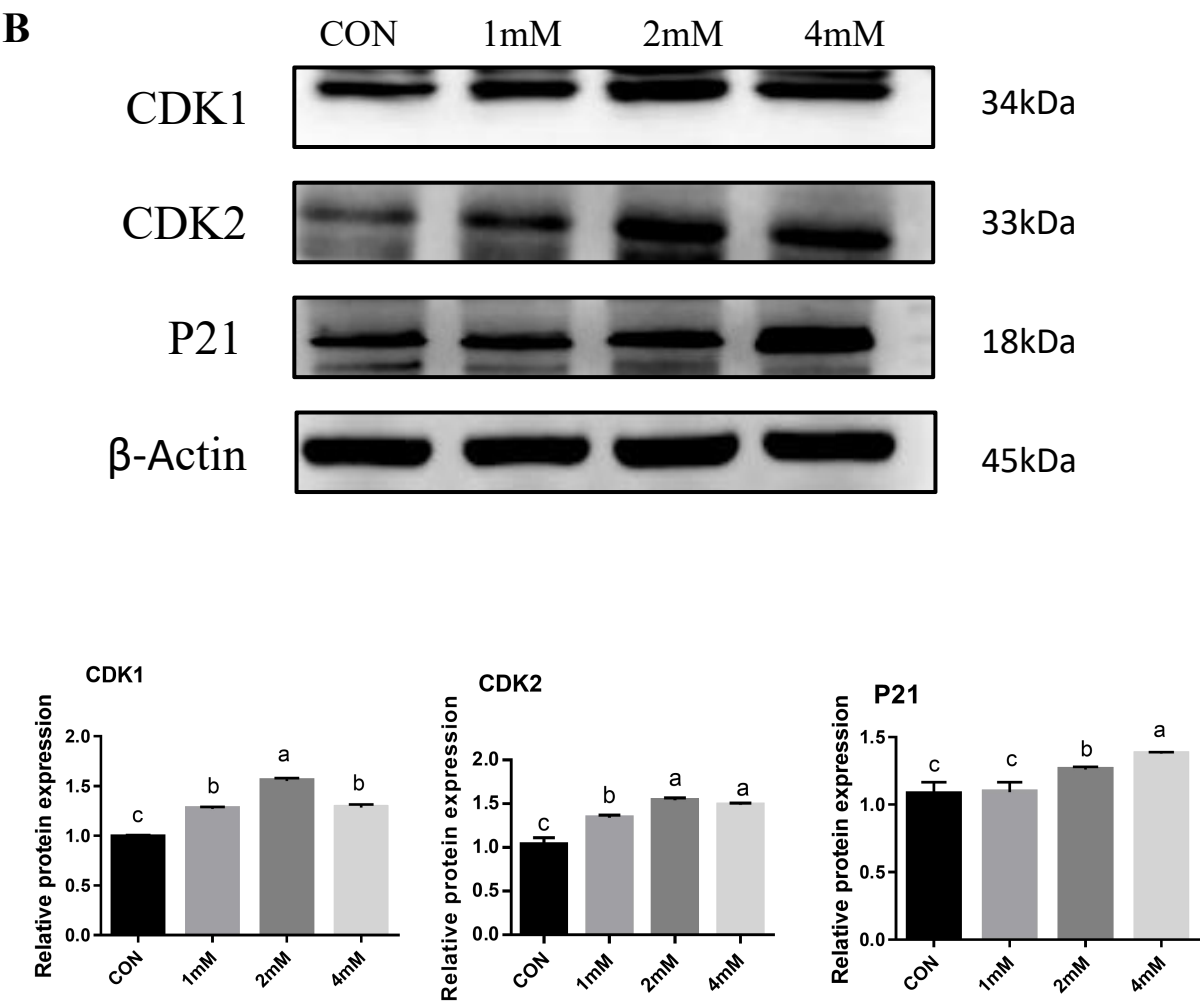

Supplement: Supplementary file 2 [file ab-24-0776-Supplementary-2.pdf]

Supplement 3.

CON

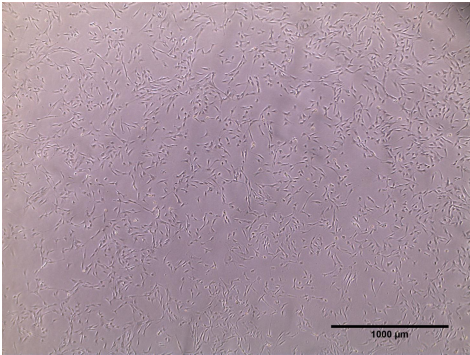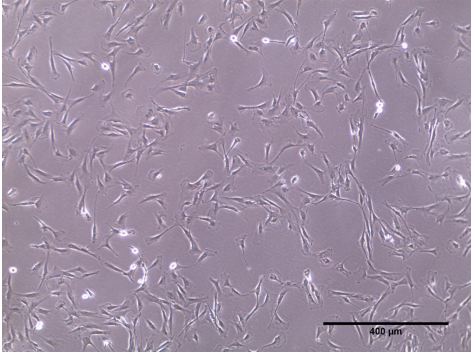

NAC

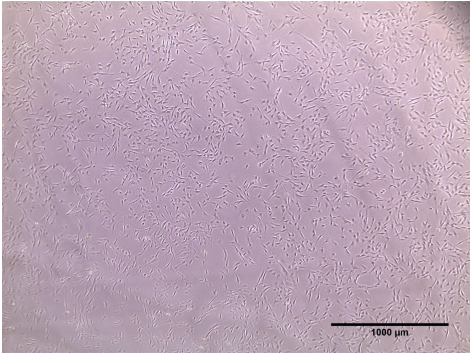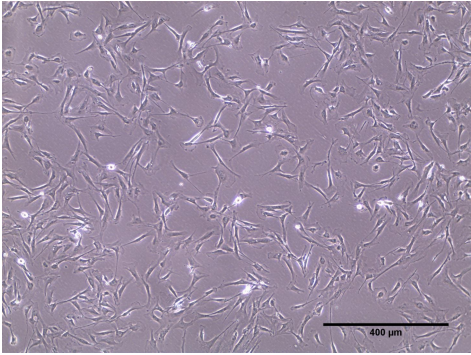

VC

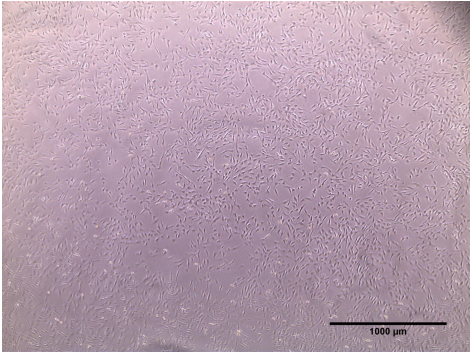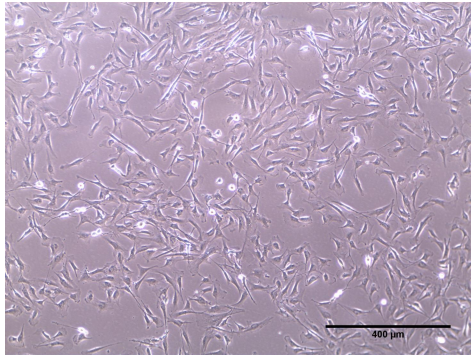

NAC+VC

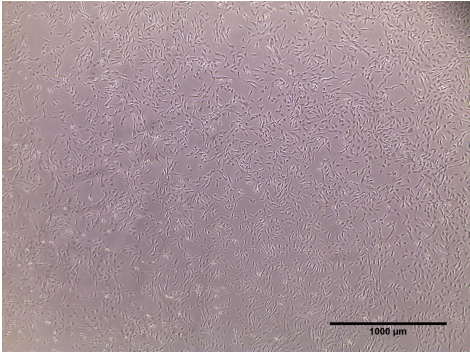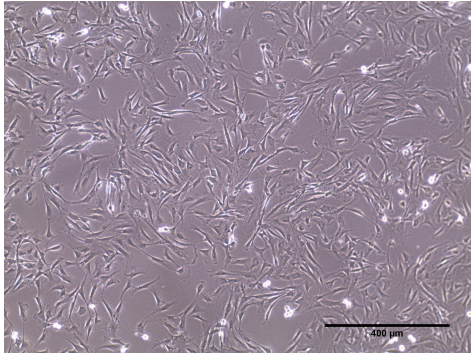

Supplement: Supplementary file 3 [file ab-24-0776-Supplementary-3.pdf]
